# Supplementary material for: Berberine Derivatives as Pseudomonas aeruginosa MexXY-OprM Inhibitors: Activity and In Silico Insights
Source: Molecules. 2021 Nov 2;26(21):6644. doi: 10.3390/molecules26216644 (PMC8587913; doi:10.3390/molecules26216644)
Supplement: Supplementary file 1 [file molecules-26-06644-s001.zip › molecules-1432191-supplementary.pdf]

Article

Berberine Derivatives as *Pseudomonas aeruginosa* MexXY-OprM Inhibitors: Activity and In Silico Insights

Giorgia Giorgini [1], Gianmarco Mangiaterra [1], Nicholas Cedraro [1], Emiliano Laudadio [2], Giulia Sabbatini [1], Mattia Cantarini [1], Cristina Minnelli [1], Giovanna Mobbili [1], Emanuela Frangipani [3], Francesca Biavasco [1] and Roberta Galeazzi [1,*]

[1] Department of Life and Environmental Sciences, Polytechnic University of Marche, via Brecce Bianche, 60131 Ancona, Italy; giorgia.giorgini@pm.univpm.it (G.G.); g.mangiaterra@staff.univpm.it (G.M.); n.cedraro@staff.univpm.it (N.C.); giulia.sabbatini@univpm.it (G.S.); m.cantarini@pm.univpm.it (M.C.); c.minnelli@staff.univpm.it (C.M.); g.mobbili@staff.univpm.it (G.M.); f.biavasco@univpm.it (F.B.)

[2] Department of Materials, Environmental Sciences and Urban Planning, Polytechnic University of Marche, via Brecce Bianche, 60131 Ancona, Italy; e.laudadio@staff.univpm.it (E.L)

[3] Department of Biomolecular Sciences, University of Urbino Carlo Bo, 61029 Urbino, Italy; emanuela.frangipani@uniurb.it

* Correspondence: r.galeazzi@staff.univpm.it

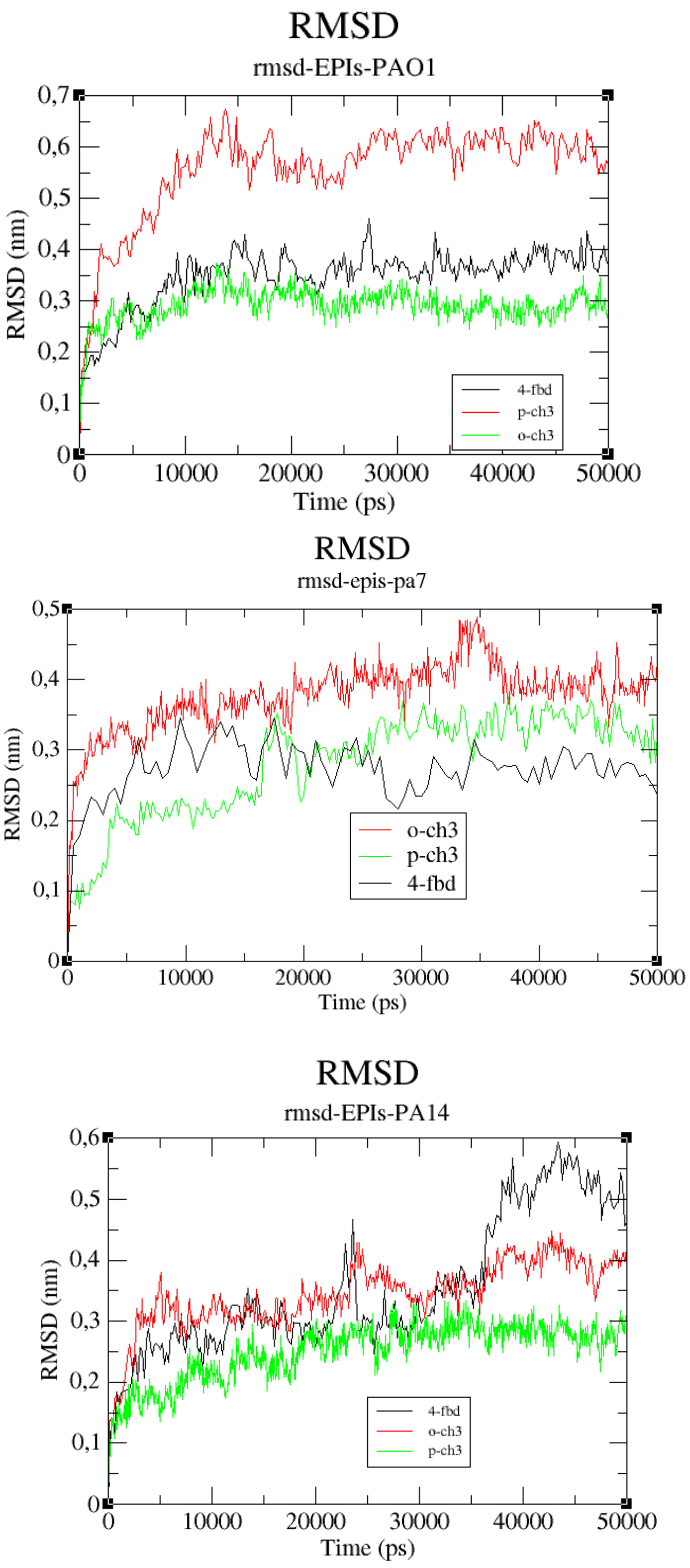

Figure S1. Calculated RMSD along the MD trajectories for the three berberine derivatives o-CH_3, p-CH_3 and p-CF_3 /mexY complexes (PAO1, PA7 and PA14).
